# Structural and functional characterisation of the entry point to pyocyanin biosynthesis in *Pseudomonas aeruginosa* defines a new 3-deoxy-d-arabino-heptulosonate 7-phosphate synthase subclass

**DOI:** 10.1042/BSR20181605

**Published:** 2018-10-15

**Authors:** Oliver W. Sterritt, Eric J.M. Lang, Sarah A. Kessans, Timothy M. Ryan, Borries Demeler, Geoffrey B. Jameson, Emily J. Parker

**Affiliations:** 1 Biomolecular Interaction Centre and School of Physical and Chemical Sciences, University of Canterbury, Christchurch 8041, New Zealand; 2 Maurice Wilkins Centre for Molecular Biodiscovery, University of Auckland, Auckland 1142, New Zealand; 3 SAXS/WAXS, Australian Synchrotron/ANSTO, 800 Blackburn Road, Clayton, VIC 3168, Australia; 4 Department of Chemistry and Biochemistry, University of Lethbridge, Alberta T1K 3M4, Canada; 5 Institute of Fundamental Sciences and the Riddet Institute, Massey University, Palmerston North 4442, New Zealand; 6 Ferrier Research Institute, Victoria University of Wellington, Wellington 6140, New Zealand

**Keywords:** aromatic amino acid, DAHP synthase, pyocyanin, shikimate

## Abstract

In *Pseudomonas aeruginosa* (*Pae*), the shikimate pathway end product, chorismate, serves as the last common precursor for the biosynthesis of both primary aromatic metabolites, including phenylalanine, tyrosine and tryptophan, and secondary aromatic metabolites, including phenazine-1-carboxylic acid (PCA) and pyocyanin (PYO). The enzyme 3-deoxy-d-*arabino*-heptulosonate 7-phosphate synthase (DAH7PS) catalyses the first committed step of the shikimate pathway, en route to chorismate. *P. aeruginosa* expresses multiple, distinct DAH7PSs that are associated with either primary or secondary aromatic compound biosynthesis. Here we report the structure of a type II DAH7PS, encoded by *phzC* as part of the duplicated phenazine biosynthetic cluster, from *P. aeruginosa* (PAO1) revealing for the first time the structure of a type II DAH7PS involved in secondary metabolism. The omission of the structural elements α_2a_ and α_2b_, relative to other characterised type II DAH7PSs, leads to the formation of an alternative, dimeric, solution-state structure for this type II DAH7PS with an oligomeric interface that has not previously been characterised and that does not facilitate the formation of aromatic amino acid allosteric binding sites. The sequence similarity and, in particular, the common N-terminal extension suggest a common origin for the type II DAH7PSs from *P. aeruginosa.* The results described in the present study support an expanded classification of the type II DAH7PSs as type II_A_ and type II_B_ based on sequence characteristics, structure and function of the resultant proteins, and on defined physiological roles within primary or secondary metabolism.

## Introduction

*Pseudomonas aeruginosa* (*Pae*) is an opportunistic human pathogen often associated with the chronic infection of patients suffering from cystic fibrosis [1]. *P. aeruginosa* produces a number of virulence factors [2] that are involved in bacterial quorum sensing [3] and establishing long-term infections, particularly of the lungs. Pyocyanin (PYO) is a secondary metabolite derived from phenazine-1-carboxylic acid (PCA) that has been shown to interfere with a number of cellular processes [4–7] and is an essential virulence factor required for pathogenic infection [8].

**Figure 1:**
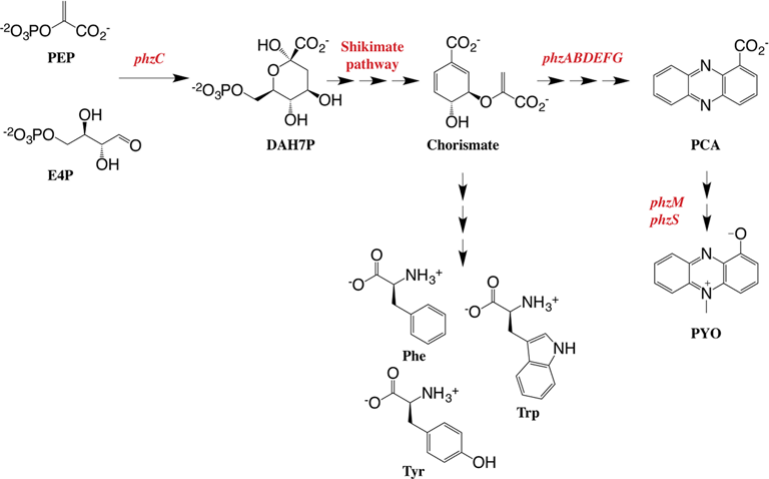
The shikimate pathway is responsible for the formation of aromatic compounds in microorganisms and plants In case of *P. aeruginosa*, the pathway end product, chorismate, is the last common precursor for the biosynthesis of both primary and secondary aromatic metabolites.

In *P. aeruginosa*, the shikimate pathway end product, chorismate, acts as the last common precursor for both primary aromatic metabolism, for the biosynthesis of the aromatic amino acids phenylalanine (Phe), tyrosine (Tyr) and tryptophan (Trp), and secondary aromatic metabolism including the biosynthesis of PCA and PYO. Starting from chorismate, the first steps of PYO biosynthesis are carried out by the duplicated seven-gene operon *phzABCDEFG* [9], leading to the formation of PCA. Expression of the *phzA-G* operon is under genetic control by the LysR-like transcriptional regulator mvfR [10,11] as part of the *P. aeruginosa* quorum-sensing circuitry. PCA is converted into PYO through additional two steps (*phzM* and *phzS*) (Figure 1) [12].

The enzymes of the *phzA–G* biosynthetic operon, along with the enzymes required for the biosynthesis of PYO from PCA, have been well characterised [13–19], with the exception of the enzyme derived from *phzC. PhzC* encodes a putative 3-deoxy-d-*arabino*-heptulosonate 7-phosphate (DAH7P) synthase (DAH7PS), which catalyses the aldol-like condensation reaction between phosphoenolpyruvate (PEP) and erythrose 4-phosphate (E4P) to form DAH7P as the first committed step of the shikimate pathway, en route to chorismate.

DAH7PSs have been classified into three broad groupings based on enzyme sequence: type Iα, type Iβ and type II [20,21]. Although less than 10% sequence identity exists between the type I and II DAH7PS groupings, all characterised examples of DAH7PSs share a common (β/α)_8_-barrel fold, a common divalent metal-ion binding site and conservation of almost all of the residues involved with E4P and PEP binding [22–33]. Various structural elements, additional to the core catalytic barrel, are associated with a diverse set of allosteric responses and the formation of alternate quaternary assemblies. The nature and location of these additional structural elements within the core catalytic barrel is characteristic of each group of DAH7PS enzymes.

While the characteristics of many examples of type I DAH7PSs have been reported, characterisation of the type II DAH7PSs has focused primarily on a group of type II enzymes that, relative to the minimalist type Iβ unadorned catalytic barrels such as *Pyrococcus furiosus* DAH7PS [25], contain both an approximately 75-residue N-terminal extension (typically providing elements β_0_, α_0a_, α_0b_ and α_0c_) and an approximately 60-residue extension to loop α_2_β_3_ (typically providing elements α_2a_ and α_2b_). For example, *Mycobacterium tuberculosis* (*Mtu*) expresses a single type II DAH7PS (*Mtu*DAH7PS), which contains these accessory structural elements. The extra-barrel elements in *Mtu*DAH7PS provide three distinct allosteric binding sites, on the single enzyme, that are each selective for either Trp, Tyr or Phe, and together they contribute towards a complex allosteric regulatory mechanism where binary or ternary combinations of aromatic amino acids that include Trp act synergistically to inhibit the enzyme [34–36]. These extensions are also responsible for the formation of the oligomeric interfaces that are present in the homotetrameric assemblies of the characterised type II enzymes. The allosteric functionality of either *Mtu*DAH7PS or the type II DAH7PS from *Corynebacterium glutamicum* (*Cgl*DAH7PS) is extended by the formation of a non-covalent complex with the AroQ_δ_ subclass of chorismate mutase (*Mtu*CM or *Cgl*CM respectively). The formation of this non-covalent complex results in an activity boost for the CM while allowing the CM to access and utilise the allosteric machinery located on the DAH7PS [32,37,38].

In comparison, *P. aeruginosa* expresses two type Iα and two type II DAH7PSs. The type II DAH7PSs are encoded by the ORFs PA1901 (and duplicated as PA4212) and PA2843 (*Pae*DAH7PS^PA1901^ and *Pae*DAH7PS^PA2843^ respectively). The structure and properties of *Pae*DAH7PS^PA2843^ have recently been reported [33] and show that *Pae*DAH7PS^PA2843^ contains an N-terminal extension that is 19 residues shorter in sequence length and has similar inserted α_2a_ and α_2b_ helices, as compared with *Mtu*DAH7PS or *Cgl*DAH7PS. Although the quaternary assemblies of *Mtu*DAH7PS and *Pae*DAH7PS^PA2843^ resemble each other, there are some differences in orientation of the extra-barrel elements within the tetramer, such that *Pae*DAH7PS^PA2843^ is inhibited by only Trp and is unaffected by combinations of Trp with Phe and Tyr due to the abbreviated N-terminal extension.

However, based on sequence alignments, there appears to be a second distinct grouping of type II DAH7PSs [39]. These DAH7PSs appear to contain an N-terminal extension to the core (β/α)_8_-barrel, comparable with that observed in *Pae*DAH7PS^PA2843^, but lack sequence corresponding to the inserted helices α_2a_ and α_2b_ for *Pae*DAH7PS^PA2843^. This difference in accessory structural elements may therefore have consequences for the formation of the quaternary assembly and hence for allosteric functionality. For example, *Pae*DAH7PS^PA1901^, found as a part of the *phzA–G* PCA biosynthetic cluster, is 43 amino acids shorter in sequence length relative to *Pae*DAH7PS^PA2843^, which is not found within the *phzA–G* PCA biosynthetic cluster. A primary structure alignment among type II DAH7PSs (Supplementary Figure S1) indicates that *Pae*DAH7PS^PA1901^ appears to lack much of the sequence that corresponds to the α_2a_ and α_2b_ helices of *Pae*DAH7PS^PA2843^, *Mtu*DAH7PS or *Cgl*DAH7PS, and hence belongs to this structurally and functionally uncharacterised second group of type II DAH7PSs.

Here we report the structure of *Pae*DAH7PS^PA1901^, characterising for the first time a short-form type II DAH7PS enzyme that is involved in secondary metabolism. The absence of the α_2a_ and α_2b_ helices relates directly to the formation of a novel, dimeric, solution-state structure for this type II DAH7PSs with altered allosteric functionality, in-line with the enzyme’s role within secondary metabolism. The structure and properties of *Pae*DAH7PS^PA1901^, in combination with those recently reported for *Pae*DAH7PS^PA2843^ [33], indicate that the evolutionary trajectory for the type II DAH7PSs may have diverged to deliver type II enzymes that function within either primary or secondary metabolism. The distinct structural and functional properties of *Pae*DAH7PS^PA1901^, in combination with sequence characteristics of the type II DAH7PSs, suggest that the type II DAH7PSs be further classified into two groups noted here as type II_A_ and type II_B_.

## Experimental procedures

### Sequence clustering analysis

Sequences of type II DAH7Ps were extracted from the Pfam database [40] (seed sequences of DAHP synthetase II - PF01474), aligned with Clustal Ω [41] and submitted to jackhmmer (part of the HMMER web server [42]) to create a hidden Markov model (HMM) profile used to scan, using an E-value sequence cut-off of 10^−15^, four different databases: UniProt [43], RefSeq [44], Pfamseq [40] and NR [44]. The E-value cut-off was carefully chosen to ensure that no type I sequences were retrieved but all type II sequences would be. This was possible due to the low sequence similarity between type I and II DAH7PSs, which is much lower compared with the sequence similarity between the two identified clusters of type II DAH7PSs. Therefore, selecting an E-value cut-off at the limit of inclusion of type I sequences enabled us to retrieve all the type II sequences available in those databases, at the time of the analysis. For each database, the scan was iterated until convergence (i.e., no new sequences identified) and the results grouped together and duplicates removed using Jalview [45], leading to 2678 non-redundant type II DAH7PS sequences which were used as an input for the clustering method implemented in CLANS [46]. Specifically, after an all-against-all BLAST search of the sequences, a force-directed pairwise similarities clustering algorithm was run for more than 500 iteration cycles at a *P*-value of 10^−15^.

### Protein expression and purification

The ORF encoding *Pae*DAH7PS^PA1901^ (EC 2.5.1.54) was amplified from *P. aeruginosa* PAO1 gDNA using the PCR. The resultant PCR product was cloned into the expression vector pET-28a(+) and engineered to incorporate an N-terminal tobacco etch virus (TEV) protease-cleavable His_6_ purification tag. The complete plasmid was sequence-verified (Macrogen), transformed into *Escherichia coli* BL21*(DE3) cells and co-expressed with the chaperonins pGroES and pGroEL. Expression was achieved following the addition of 1 mM IPTG and subsequent incubation at 23°C for 16 h. Cells were harvested by centrifugation (12000 ***g***, 15 min). Cell lysis was achieved in lysis buffer (10 mM bis-tris propane pH 8.0, 200 mM KCl, 1 mM tris(2-carboxyethyl)phosphine hydrochloride, 200 μM PEP, 10 mM imidazole) by sonication (4 × 5-min cycles at 80% power). Cellular DNA was degraded by the addition of benzonase before the removal of cellular debris by centrifugation (40000 ***g***, 30 min). Purification was carried out using Co^2+^ affinity chromatography, incubation with TEV protease (4°C, 3 h), and size-exclusion chromatography. In brief, the soluble fraction of the cell lysate (containing PaeDAH7PS^PA1901^) was loaded on to a talon trap column pre-equilibrated with lysis buffer. Contaminating *E. coli* proteins were washed through the column before isocratic elution of PaeDAH7PS^PA1901^ in buffer containing 10 mM bis-tris propane pH 8.0, 200 mM KCl, 1 mM tris(2-carboxyethyl)phosphine hydrochloride, 200 μM PEP, 100 mM imidazole. Protein samples were diluted (1:1) with lysis buffer immediately after elution from the column. The His_6_ purification tag was cleaved by incubation with TEV protease (2 mg, 4°C, 3 h) before the cleaved tag was removed from the protein sample by a second round of affinity purification. Protein samples were concentrated and loaded on to a Hiload™ 26/30 Superdex™ 200 column pre-equilibrated with buffer containing 10 mM bis-tris propane pH 8.0, 200 mM KCl, 1 mM tris(2-carboxyethyl)phosphine hydrochloride, 200 μM PEP. Protein concentrations were determined using a Nanodrop ND-1000 spectrophotometer, at 280 nm, using the molar extinction coefficient (54430 M^−1^.cm^−1^) calculated for the protein using ProtParam. Purified protein samples were flash frozen in liquid nitrogen and stored at −80°C.

### MS

The molecular weight of *Pae*DAH7PS^PA1901^ was determined by ESI MS (Bruker maXis 3G). Protein samples were dialysed into Milli-Q water and diluted to a concentration of 0.3 mg.ml^−1^ prior to analysis. The molecular mass of a single chain of *Pae*DAH7PS^PA1901^ was found to be 44470 Da compared with the calculated theoretical mass of 44468 Da (ProtParam).

### Enzyme kinetic assays

The activity of *Pae*DAH7PS^PA1901^ was monitored over a range of temperatures (from 35 to 50°C) and a range of pHs (pH 6.5–8.5) based on methods previously described [26] using a Varian Cary 300 UV-Vis spectrophotometer. Metal ion dependency was determined by monitoring the activity of *Pae*DAH7PS^PA1901^ in the presence of 100 μM of various divalent metal cations. The enzyme was pre-treated with EDTA (0.5 mM, 2 h) to remove background metal ions before being buffer-exchanged into assay buffer that had been pre-treated with Chelex (Bio-Rad). PEP (Sigma) and E4P (Sigma) concentrations were held constant at 150 μM, except when determining the respective *K*_M_ values, determined by monitoring the activity of *Pae*DAH7PS^PA1901^ in the presence of 10–200 μM (E4P) or 10–400 μM (PEP) of the substrate for which *K*_M_ was being measured. For the inhibition studies, stock solutions of either Trp, Tyr or Phe were prepared in ultrapure water. Stock solutions of phenazine or PCA were prepared in DMSO and activity was compared with controls where phenazine or PCA was substituted for an equivalent amount of DMSO. All reactions were carried out in the presence of 100 μM Co^2+^, except when determining metal ion preference, and the reaction was initiated by the addition of purified *Pae*DAH7PS^PA1901^. Initial reaction rates were determined using a least-squares fit of the data.

### Analytical ultracentrifugation

Sedimentation velocity experiments were performed in a Beckman Coulter Model XL-I analytical ultracentrifuge equipped with UV/Vis scanning optics. Reference buffer solution (50 mM bis-tris propane, pH 7.5, 200 mM KCl, 100 μM cobalt chloride, 200 μM PEP) and sample solutions (including reference buffer solution with *Pae*DAH7PS^PA1901^ at three concentrations: 0.34 mg.ml^−1^ (8 μM), 1.0 mg.ml^−1^ (23 μM), and 1.35 mg.ml^−1^ (30 μM)) were loaded into 12-mm double-sector cells with standard Epon 2-channel centerpieces and sapphire windows. For the two higher concentrations (23 and 30 μM), cells were mounted in an eight-hole An-50 Ti rotor and centrifuged at 50000 rpm at 20°C, with absorbance measurements at a wavelength of 295 nm (collected in intensity mode) recorded over a radial position range of 5.8–7.3 cm within the cells taken at sediment boundary intervals of 0.003 cm. In order to gain a more optimal signal-to-noise ratio for the lowest concentration (8 μM) and buffer without protein present, cells were mounted in a four-hole An-60 Ti rotor and centrifuged at 40000 rpm at 20°C, with absorbance measurements at a wavelength of 240 nm (collected in intensity mode) recorded over a radial position range of 5.8–7.3 cm within the cells taken at sediment boundary intervals of 0.003 cm. Further sedimentation velocity experiments, utilising protein at 17 μM, in the presence or absence of 200 μM of either PYO, Phe, Tyr or Trp, were carried out using an eight-hole An-50 Ti rotor and centrifuged at 35000 rpm at 20°C, with absorbance measurements at a wavelength of 290 nm recorded over a radial position range of 5.8–7.3 cm within the cell taken at sediment boundary intervals of 0.003 cm. Buffer density (1.0129 g/ml) and buffer viscosity (1.050 cP) were experimentally measured with an Anton Paar DMA4100M density meter and Anton Paar Lovis 2000 ME microviscometer respectively. The 2DSA-Monte Carlo, van Holde-Weischet, and Discrete Model Genetic Algorithm (DMGA) analyses were performed using UltraScan III [47–50]. Bead modelling and hydrodynamic calculations were performed using UltraScan Solution Modeller (US-SOMO) [51,52].

### Small angle X-ray scattering data collection and analysis

Size-exclusion chromatography coupled small angle X-ray scattering (SEC-SAXS) data were collected at the SAXS/WAXS beamline at the Australian Synchrotron [53] using a sheath flow sample environment [54] at 12 keV (1.0332 Å), using a detector distance of 1600 mm, and at a temperature of 293 K. Data were collected immediately after elution from a Superdex S200 (5 × 150 mm) column at a flow rate of 0.2 ml.min^−1^ [55]. Samples were loaded on to the column at protein concentrations of 8.0, 5.0 and 1.0 mg.ml^−1^ in buffer containing 50 mM bis-tris propane pH 7.5, 100 μM cobalt chloride, 200 μM PEP, 5% glycerol.

Data were processed using the reduction software *ScatterBrain 2.83*, developed at the Australian Synchrotron. Scattering intensity (*I*) was plotted versus *q*, as a log-linear plot, and analysed using the *ATSAS* package [56]. Deconvolution of the data was achieved using the HPLC module of the *SOMO* package [52,57] by fitting two pure Gaussian functions to each SEC-SAXS dataset. *GASBOR* [58] was used to generate *ab initio* dummy residue models from the *P*(*r*) obtained from the deconvoluted data for peaks A and B, which were overlaid with the crystal structure of *Pae*DAH7PS^PA1901^ (Protein Data Bank (PDB): 6BMC).

### Crystallography and structure determination

Protein crystals were prepared, by microbatch crystallisation [59], by mixing equal volumes of purified protein (final protein concentration 3–5 mg.ml^−1^ (67–112 μM)) with reservoir solution (0.2 M sodium fluoride, 1 mM cobalt chloride, 1 mM PEP, 18% PEG 3350) and incubating at 278 K for 1–2 days. Crystals were flash frozen at 110 K in cryoprotectant containing 25% glycerol and mother liquor. X-ray diffraction data were collected at the Australian Synchrotron using the MX2 beamline [60], equipped with an Eiger 16M detector, at a wavelength of 0.9536 Å. Diffraction data were processed using XDS [61] and AIMLESS [62], and the structure of *Pae*DAH7PS^PA1901^ was solved by molecular replacement (MOLREP) [63] using a single chain of *Pae*DAH7PS^PA2843^ (PDB: 5UXM) [33] as the search model. All ligands and waters were removed from the search model prior to molecular replacement, as were residues corresponding to the inserted helices α_2a_ and α_2b_. The sequence identity between the search model and the target protein was 43%. The model was built using COOT [64] and refined with REFMAC [65].

### Interface analysis

PISA [66] was used to visualise and examine the residues involved in interface formation. LSQKAB [67] was used to superpose and compare the structures.

### PDB accession codes

Atomic co-ordinates and structure factors for the structure described in this work have been deposited in the PDB with the accession code 6BMC.

## Results and discussion

### Clustering of type II DAH7PS sequences reveals an uncharacterised subgroup of type II enzymes

**Figure 2:**
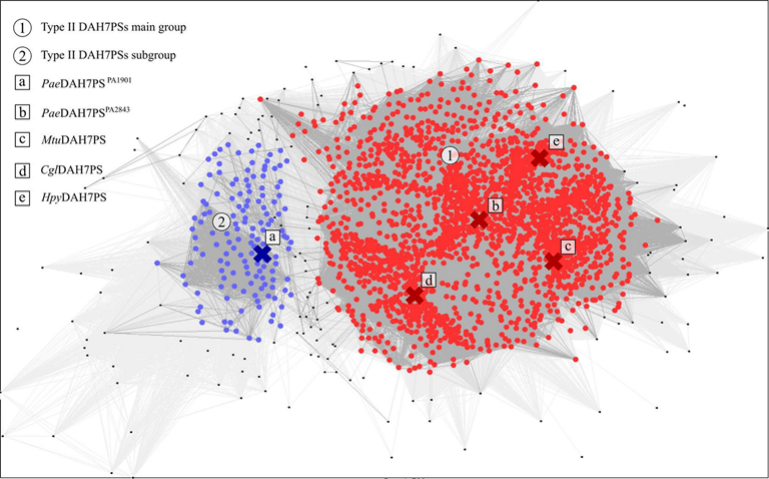
CLANS clustering analysis of type II DAH7PS sequences reveals two distinct groups of type II DAH7PSs Each dot represents a type II DAH7PS sequence. The main group of type II DAH7PSs (1) is indicated by the red dots. The second group of type II DAH7PSs (2) is indicated by the blue dots. Lines connecting the dots indicate the sequence similarity relationship at the BLAST *P*-value cut-off of 10^−50^, the darker the colour, the higher the sequence similarity. Crosses marked (a–e) correspond to the sequences of *Pae*DAH7PS^PA1901^, *Pae*DAH7PS^PA2843^, *Mtu*DAH7PS, *Cgl*DAH7PS and *Helicobacter pylori* DAH7PS (*Hpy*DAH7PS) respectively.

Clustering of type II DAH7PSs, based on pairwise sequence similarity, enables the identification of two main clusters of sequences presenting high intra- and low inter-cluster sequence similarity (Figure 2). The main cluster contains sequences corresponding to full-length type II DAH7PSs (including *Pae*DAH7PS^PA2843^, *Mtu*DAH7PS and *Cgl*DAH7PS) that contain both an N-terminal extension and the α_2a_ and α_2b_ inserted helices. However, a second distinct group of sequences, which are distant from the main cluster, is also evident. Sequences from this second grouping (of which *Pae*DAH7PS^PA1901^ is a member) are shorter in sequence length, relative to those found in the main type II DAH7PS cluster, due to the predicted omission of the sequence corresponding to the α_2a_ and α_2b_ helices. Although there is high sequence homology among members of each subgrouping (for example, *Pae*DAH7PS^PA2843^ and *Mtu*DAH7PS share 50% sequence identity and 67% sequence similarity), a comparison between sequences from the main cluster with those from the subgroup reveals increased sequence diversity between the two type II DAH7PS groups. For example, *Pae*DAH7PS^PA1901^ and *Mtu*DAH7PS share only 38.5% sequence identity and 50.0% sequence similarity, and *Pae*DAH7PS^PA1901^ and *Pae*DAH7PS^PA2843^ share 38.4% sequence identity and 52.0% sequence similarity. Does this difference in sequence characteristics translate to altered structural and/or functional properties for this second uncharacterised group of type II DAH7PSs, analogous to those observed for the type Iα compared with type Iβ DAH7PSs? To address this question, we sought full characterisation of *Pae*DAH7PS^PA1901^.

### *Pae*DAH7PS^PA1901^ is insensitive to aromatic amino acids or PCA

**Figure 3:**
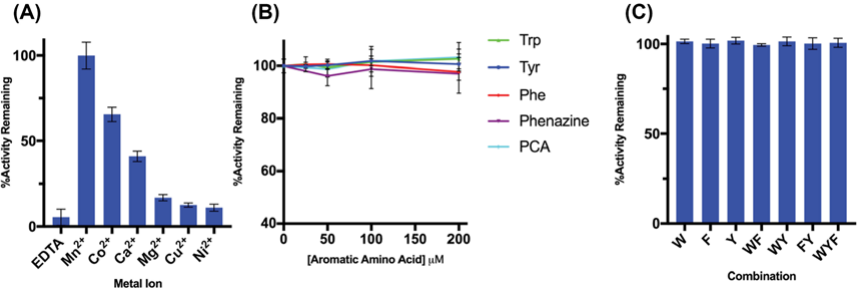
Activity of *Pae*DAH7PS^PA1901^ (**A**) In the presence of 100 μM of various divalent metal cations or 100 μM of EDTA. (**B**) In the presence of single aromatic amino acids or secondary metabolites (Trp, green; Tyr, blue; Phe, red; phenazine, purple; PCA, cyan) or (**C**) binary and ternary combinations of aromatic amino acids. Each single letter code corresponds to 100 μM of the corresponding amino acid. PEP and E4P concentrations were held constant for all measurements at 150 μM each. Error bars represent the S.D. of triplicate measurements.

The purified recombinant *Pae*DAH7PS^PA1901^ was found to be catalytically active over a range of temperatures between 35 and 50°C and over a range of pH between pH 6.5 and 7.5 (Supplementary Figure S2), in contrast with *Pae*DAH7PS^PA2843^ where maximal activity is observed over a narrow range of temperatures and pH [33]. Maximal *Pae*DAH7PS^PA1901^ activity was observed at pH 7.5 and 45°C. Metal ion preference was investigated by monitoring the activity of *Pae*DAH7PS^PA1901^ in the presence of various divalent metal cations, and it was found that Mn^2+^ was most the activating (Figure 3A). Subsequent assays were carried out at pH 7.5, 37°C in the presence of Co^2+^ in order to provide a comparison with *Pae*DAH7PS^PA2843^, which exhibits maximal activity under these conditions [33]. Apparent *K*_M_ values for *Pae*DAH7PS^PA1901^ for PEP and E4P were determined to be 17 ± 1 and 16 ± 3 μM respectively (Table 1). The Michaelis constants are in-line with other characterised type II DAH7PSs [26,33,39,68], including *Pae*DAH7PS^PA2843^, and the turnover number, *k*_cat_, for *Pae*DAH7PS^PA1901^ was determined to be 19.8 ± 0.4 s^−1^.

**Table 1:** Kinetic parameters determined for *Pae*DAH7PS^PA1901^ at pH 7.5, 37°C and comparison with those previously reported for either *Pae*DAH7PS^PA2843^ or *Mtu*DAH7PS

Enzyme	Metal ion	*k*_cat_ (s^−1^)	*K*_M_^E4P^ (μM)	*K*_M_^PEP^ (μM)	*k*_cat_/*K*_M_^E4P^ (s^−1^.μM^−1^)	*k*_cat_/*K*_M_^PEP^ (s^−1^.μM^−1^)
*Pae*DAH7PS^PA1901^	Co^2+^	19.8 ± 0.4	16 ± 3	17 ± 1	1.24 ± 0.15	1.16 ± 0.10
*Pae*DAH7PS^PA2843^ [33]	Co^2+^	40.0 ± 0.7	18 ± 1	28 ± 1	2.20 ± 0.20	1.40 ± 0.10
*Mtu*DAH7PS [26]	Mn^2+^	3.1 ± 0.1	25 ± 1	37 ± 3	0.12 ± 0.10	0.08 ± 0.01

The activity of *Pae*DAH7PS^PA1901^ was monitored in the presence of increasing concentrations of the aromatic amino acids Trp, Tyr, Phe or the secondary metabolites phenazine or PCA. At concentrations up to 200 μM Trp, Tyr, Phe, phenazine or PCA, *Pae*DAH7PS^PA1901^ activity was found to be comparable with that observed in the absence of aromatic amino acids or secondary metabolites, analogous to the allosteric behaviour of the unregulated type Iβ DAH7PSs [69] (Figure 3B,C). Combinations of aromatic amino acids appear to have no inhibitory effect on *Pae*DAH7PS^PA1901^ activity similar to that observed in the absence of aromatic amino acids (Supplementary Figure S3). The observed absence of allosteric sensitivity in *Pae*DAH7PS^PA1901^ is in contrast with *Mtu*DAH7PS or *Pae*DAH7PS^PA2843^ where allosteric inhibition was observed under the same conditions that were used to evaluate the allosteric properties of *Pae*DAH7PS^PA1901^. In particular, in *Mtu*DAH7PS, any binary or ternary combination of aromatic amino acids that includes Trp acts to synergistically inhibit the enzyme [34–36] or, in *Pae*DAH7PS^PA2843^, sensitivity to Trp alone was observed, but this sensitivity was diminished in comparison with that observed for *Mtu*DAH7PS [33].

### The crystal structure of *Pae*DAH7PS^PA1901^ reveals novel quaternary assembly

**Figure 4:**
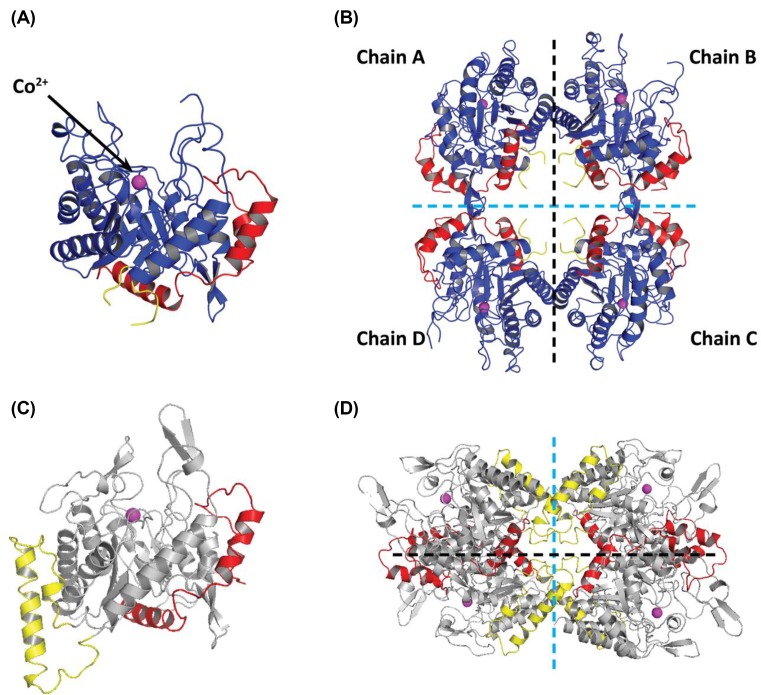
Comparison among the crystal structures of *Pae*DAH7PS^PA1901^ and *Pae*DAH7PS^PA2843^ (**A**) A single chain of *Pae*DAH7PS^PA1901^ (PDB: 6BMC). The core (βα)_8_ catalytic domain is shown in blue, the N-terminal extension (residues 1–59) is shown in red, the short extension to loop α_2_β_3_ (residues 167–181) is shown in yellow (residues 172–177 are unresolved in this structure), and the active site Co^2+^ ion is shown in magenta. (**B**) The homotetrameric structure of *Pae*DAH7PS^PA1901^ as observed in the crystal structure. The major interface (indicated by the black dashed line) is formed primarily by residues located on helices α_1_ and α_8_. The minor interface (indicated by the cyan dashed line) is formed by residues located on loop α_3_β_4_. (**C**) A single chain of *Pae*DAH7PS^PA2843^ (PDB: 5UXM) shown in grey with the N-terminal extension (residues 1–59) shown in red, the inserted helices α_2a_ and α_2b_ (residues 179–225) shown in yellow and the active site Co^2+^ ion shown in magenta. (**D**) The homotetrameric structure of *Pae*DAH7PS^PA2843^. The major interface (indicated by the black dashed line) is formed primarily by residues located on helix α_2_. The minor interface (indicated by the cyan dashed line) is formed by residues primarily located on helix α_2b_.

The crystal structure of *Pae*DAH7PS^PA1901^ (phzC) was solved (resolution 2.70 Å, *R_free_* = 0.280) in complex with the substrate PEP and a Co^2+^ ion, with attached water molecule, bound at the active site, revealing for the first time the structure of a short-form type II DAH7PS that is involved in secondary (here phenazine) metabolism. *Pae*DAH7PS^PA1901^ crystallised in the space group *C*222_1_, with two DAH7PS chains present in the asymmetric unit. Application of a two-fold crystallographic symmetry operation results in the assembly of a homotetrameric species, which comprises both a major and minor interfaces. Chain A residues 119–123, 172–177 and 389–405, and chain B residues 121–123, 170–177 and 389–405 are not resolved in this structure and were therefore not included in the final model (Figure 4). Data collection and refinement statistics are shown in Table 2.

**Table 2:** Data collection and refinement statistics for the crystal structure determined for *Pae*DAH7PS^PA1901^

**PDB code**	6BMC
**Space group**	*C*222_1_
**Data collection and processing statistics**	
Unit cell dimensions (Å)	
* a*	53.8
* b*	169.7
* c*	170.6
Resolution range (outer shell) (Å)	49.08–2.70 (2.83–2.70)
Wavelength (Å)	0.9536
Unique reflections	21959 (2879)
Completeness (%)	99.9 (100.0)
Redundancy	9.4 (10.0)
*I/σ*	10.6 (0.8)
*R*_merge_	0.128 (2.619)
*R*_pim_	0.064 (1.280)
*Cc*_1/2_	0.999 (0.461)
Refinement resolution (Å)	2.70 (2.83–2.70)
**Refinement statistics**	
*R* factor	0.23
*R*_free_ (5 %)	0.28
Number of non-hydrogen atoms	
Protein atoms	5667
Water atoms	13
Ligand atoms	22
Wilson *B* value (Å^2^)	73.7
Mean *B* value (Å^2^)	
All atoms	88.1
Protein atoms	91.2
Ligand atoms	65.1
Water atoms	83.1
**RMSDs from ideality**	
Bond lengths (Å)	0.004
Bond angles (°)	0.800
Residues in the most favoured region of Ramachandran plot (%)	95.45
Residues in the forbidden region of Ramachandran plot (%)	0.27^1^

1 Cys^372^ from both chains A and B.

As with all DAH7PS structures reported to date [22–33], *Pae*DAH7PS^PA1901^ features a core (β/α)_8_-barrel fold, with an N-terminal extension to the core catalytic domain consistent with its membership of the type II DAH7PS family (Figure 4). Residues 1–59 form an N-terminal extension to the barrel, providing additional helices α_0a_, α_0b_ and α_0c_, with strong structural homology to the equivalent helices in other structurally characterised type II DAH7PSs, in particular *Pae*DAH7PS^PA2843^ [33]. Residues 167–181 form loop α_2_β_3_, which lacks the inserted helices α_2a_ and α_2b_ as observed in both *Mtu*DAH7PS and *Pae*DAH7PS^PA2843^ [26,33].

**Figure 5:**
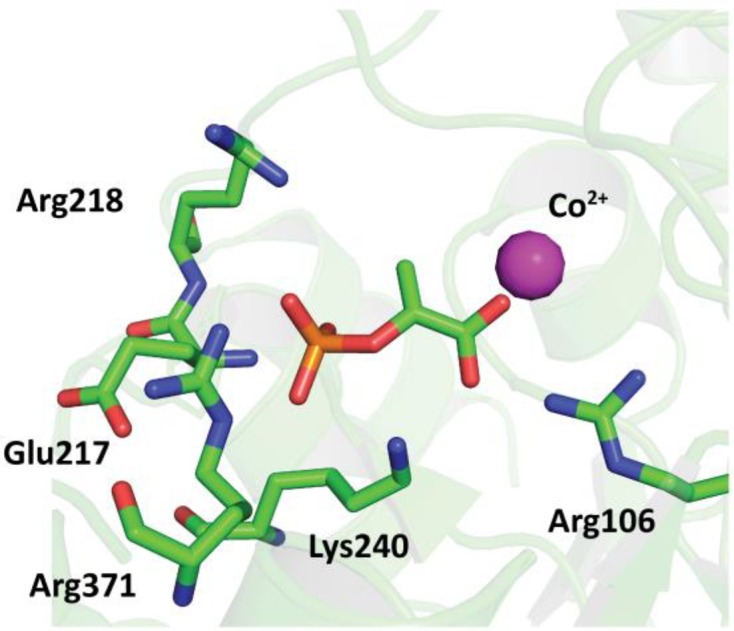
The active site of *Pae*DAH7PS^PA1901^ (PDB: 6BMC) showing the binding mode of the substrate PEP

^95^The major interface is formed by residues primarily located on helices α_4_ and α_5_ (and β-strands β_5a_ and β_5b_) for the type Iα DAH7PSs [22,30,70] or by residues primarily located on helices α_6_ and α_7_ for the type Iβ DAH7PSs [25,31] or by residues primarily located on helices α_0c_ and α_2_ for the type II DAH7PSs [26,32,33]. Based on inspection of the protein sequence, and comparison with that of *Pae*DAH7PS^PA2843^ or *Mtu*DAH7PS, we naïvely anticipated that the interface involving helices α_0c_ and α_2_ in *Pae*DAH7PS^PA2843^ or *Mtu*DAH7PS would be preserved in *Pae*DAH7PS^PA1901^. Surprisingly, this is not the case (Figure 6). The major interface for *Pae*DAH7PS^PA1901^ is instead formed through interactions between chains A and B (or chains C and D) primarily involving residues located on helices α_1_ and α_8_, assembling a completely distinct major interface compared with that observed for any of the DAH7PSs characterised to date. For *Pae*DAH7PS^PA1901^, a total of 22 residues from chain A and 23 residues from chain B are involved in the formation of this major interface, burying 840 Å^2^ (5.5%) or 819 Å^2^ (5.3%) of the surface area of each chain respectively, comparable with the surface area involved in the formation of the major interface observed for either *Pae*DAH7PS^PA2843^ or *Mtu*DAH7PS [26,32,33]. Four equivalent pairs of salt bridges were identified to form between chain A atoms Glu77_OE1 (and Glu77_OE2), Glu87_OE1 (and Glu87_OE2), Arg94_NH1 (and Arg94_NH2), Arg103_NH1 (and Arg103 NH2) and chain B atoms Arg94_NH2, Arg103_NH2, Glu77_OE1, and Glu87_OE1 respectively (Figure 7). A pair of equivalent hydrophobic contacts are made between chain A residue Leu^95^ and chain B residues Pro^348^ and Val^85^ (and *vice versa*), and between chain A residue Ala^92^ and chain B residue Leu^88^ (and *vice versa*). Hydrophobic contacts are also found between chain A residue Leu^88^ and two residues from chain B: Leu and Leu^88^ (and *vice versa*). Further hydrophobic contacts are found between chain A residues Trp^379^, Leu^382^ and chain B residues Met^386^ and Leu^382^ respectively.

The active site for *Pae*DAH7PS^PA1901^ is located at the C-terminal end of the core (βα)_8_ catalytic barrel and is comparable with that observed among the type II DAH7PSs in terms of residue identity. The PEP phosphate group is co-ordinated by atoms Glu217_N, Arg218_NH1, Arg271_NE, Arg271_NH2 and Lys240_NZ whereas the carboxylate group of PEP is co-ordinated by atoms Arg106_NH1 and Lys240_NZ (Figure 5 and Supplementary Figure S4).

**Figure 6:**
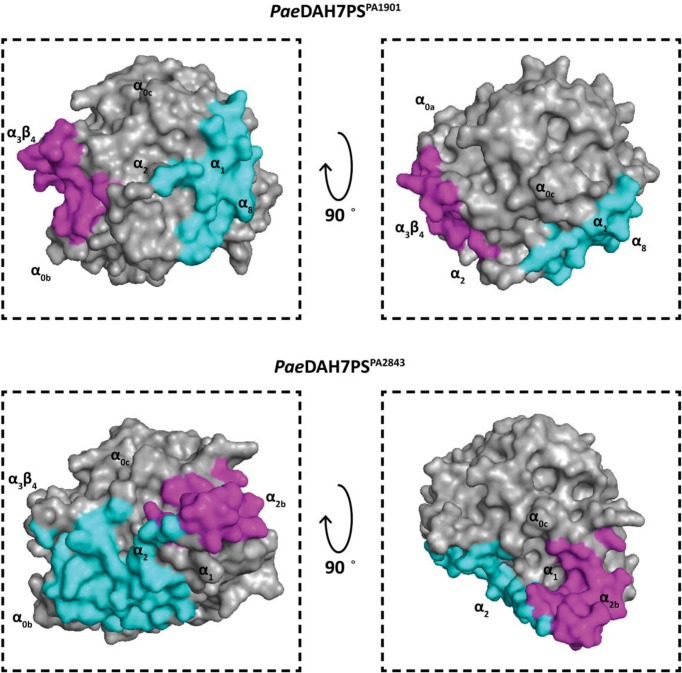
Comparison between the regions of the (β/α)_8_-barrel involved in the formation of the oligomeric interfaces in *Pae*DAH7PS^PA1901^ (PDB: 6BMC) and *Pae*DAH7PS^PA2843^ (PDB: 5UXM) For all figures, surface area involved in the formation of the major interface is highlighted in cyan and surface area involved in the formation of the minor interface is highlighted in magenta. The upper pair of figures is shown in the same orientation relative to the corresponding figure in the lower pair of figures. The approximate locations of key structural elements are indicated.

**Figure 7:**
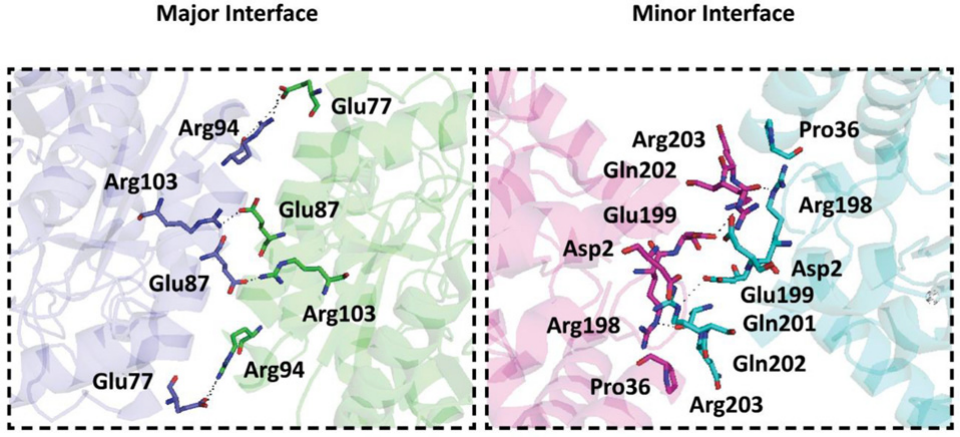
Charged residues involved in the formation of both the major and minor interface of *Pae*DAH7PS^PA1901^ For the major interface, chain A is shown in blue and chain B is shown in green. For the minor interface, chain A is shown in magenta and chain D is shown in cyan. For both figures, polar contacts are indicated by the black dashed lines.

Unexpectedly, despite the absence of the α_2a_ and α_2b_ helices in *Pae*DAH7PS^PA1901^, which are associated with the formation of the minor interface in either *Pae*DAH7PS^PA2843^ or *Mtu*DAH7PS, an alternative interaction to deliver a homotetrameric species is observed in *Pae*DAH7PS^PA1901^ (Supplementary Table S3). This alternative minor interface for *Pae*DAH7PS^PA1901^ is formed through interaction between chains A and D (or chains B and C) by residues located primarily on loop α_3_β_4_ and is distinctive from the minor interface observed for all characterised DAH7PSs (both type I and II). In comparison, the minor interface is formed by residues primarily located on helix α_0a_ for the type Iα DAH7PSs [22,30,70] or by residues located on helices α_4_ and α_5_ for the type Iβ DAH7PSs [25,31] or by residues primarily located on helices α_2a_ and α_2b_ for the type II DAH7PSs [26,32,33]. For *Pae*DAH7PS^PA1901^, a total of 20 residues each from chain A or D are involved in the formation of this minor interface with a buried surface area of 598 Å^2^ (4.0%) or 602 Å^2^ (4.0%) respectively. A pair of salt bridges is formed between chain A atom Asp2_OD1 and chain D atom Arg203_NH2 (likewise for chain A atom Arg203_NH2 and chain D atom Asp2_OD1) as well as a hydrogen bond between chain A atom Arg198_NE and chain D atom Glu201_O (likewise for chain A atom Glu201_O and chain D atom Arg198_NE). In addition, a limited suite of hydrophobic contacts is found between methylene groups of Gln^202^ and Arg^199^ in chain A and Pro^36^ and Arg^203^ in chain D (and *vice versa*).

For *Mtu*DAH7PS, three distinct aromatic amino acid allosteric binding sites exist that are each selective for either Trp, Tyr or Phe. The Phe and Trp sites are located at the oligomeric interfaces and are intimately associated with the formation of the quaternary assembly [34,36,71]. In comparison, for *Pae*DAH7PS^PA2843^ a single allosteric binding site exists at the tetramer interface that is sensitive for Trp [33] and structurally comparable with the Trp site of *Mtu*DAH7PS. For *Pae*DAH7PS^PA1901^, the alternative oligomeric interfaces and subsequent formation of a significantly different quaternary assembly, relative to either *Pae*DAH7PS^PA2843^ or *Mtu*DAH7PS, disrupts completely the formation of any aromatic amino acid allosteric binding sites that are comparable with those observed for either *Pae*DAH7PS^PA2843^ or *Mtu*DAHPS. Consistent with this is the observation made during functional characterisation that *Pae*DAH7PS^PA1901^ is insensitive to allosteric regulation by aromatic amino acids, confirming that *Pae*DAH7PS^PA1901^ functions primarily within secondary metabolism.

### Solution-state structure of *Pae*DAH7PS^PA1901^

**Figure 8:**
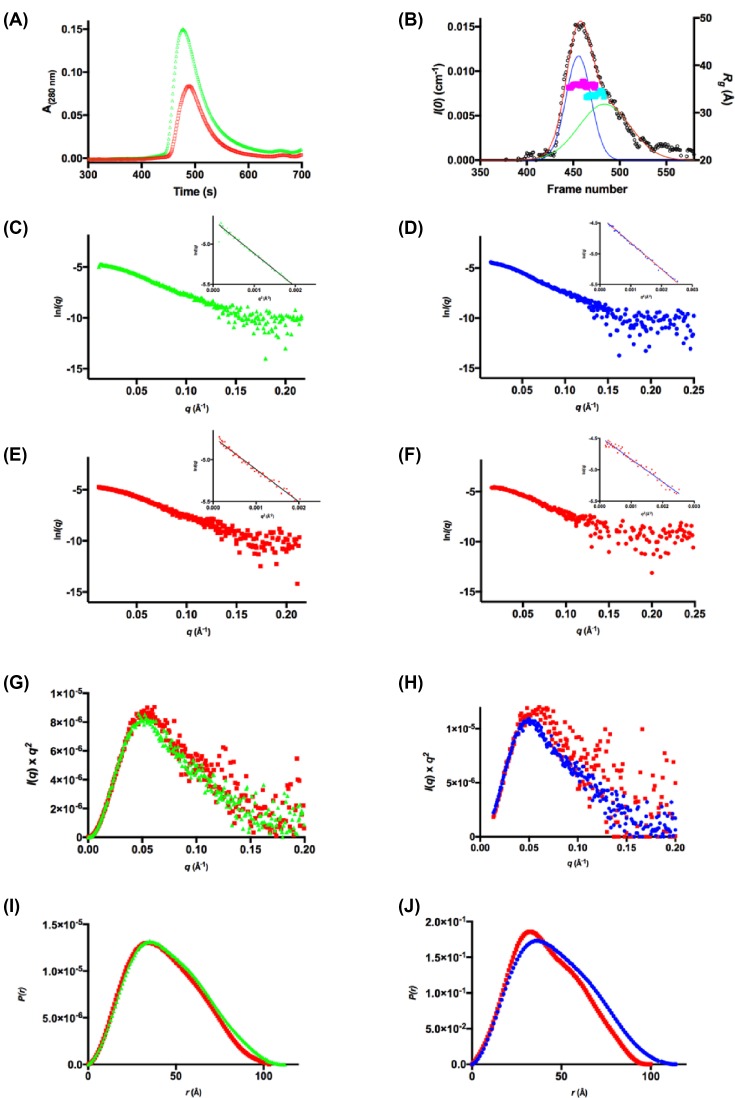
SEC-SAXS analysis for *Pae*DAH7PS^PA1901^ (**A**) SEC-SAXS elution profile for two injected enzyme concentrations (5.0 mg.ml^−1^, red squares and 8.0 mg.ml^−1^, green triangles). (**B**) Deconvolution of the SEC-SAXS data indicates two Gaussian components (peak A, blue line and peak B, green line. Sum, red line). The *R_g_* values across each peak are indicated as magenta or cyan squares respectively. (**C**) the SAXS profile for the non-deconvoluted 8.0 mg.ml^−1^. (**D**) The SAXS profile for the deconvoluted 8.0 mg.ml^−1^ peak A. (**E**) The SAXS profile for the non-deconvoluted 5.0 mg.ml^−1^. (**F**) The SAXS profiles for the deconvoluted 8.0 mg.ml^−1^ peak B. Guinier plots are inset for frames (C–F). (**G**) Kratky plots of the non-deconvoluted data in (C,E) (8.0 mg.ml^−1^, green triangles and 5.0 mg.ml^−1^, red squares). (**H**) Kratky plots of the deconvoluted data in (D,F) (peak A, blue circles and peak B, red squares). (**I**) *P*(*r*) plots for the non-deconvoluted data in (C,E) (8.0 mg.ml^−1^, green triangles and 5.0 mg.ml^−1^, red squares). (**J**) *P*(*r*) plots for the deconvoluted data in (D,F) (peak A, blue circles and peak B, red squares).

SEC-SAXS data were collected using three different starting protein concentrations: 1.0, 5.0 and 8.0 mg.ml^−1^ (22–180 μM) to investigate the solution-state structure of *Pae*DAH7PS^PA1901^ and the concentration dependency of quaternary structure (Figure 8 and Table 3, Supplementary Figure S5 and Tables S1 and S2).

**Table 3:** SEC-SAXS parameters determined for *Pae*DAH7PS^PA1901^

	Non-deconvoluted 8.0 mg.ml^−1^	8.0 mg.ml^−1^ Peak A	8.0 mg.ml^−1^ Peak B	1.0 mg.ml^−1^
**Guinier analysis**				
*R*_g_ (Å)	34.4 ± 0.6	36.0 ± 1.2	33.0 ± 1.4	34.0 ± 2
*I*(0) (cm^−1^)	0.030 ± 0.000	0.012 ± 0.000	0.012 ± 0.000	0.010 ± 0.001
*q*_min_	0.012	0.013	0.013	0.013
Correlation coefficient, *r*^2^	0.978	0.996	0.971	0.960
**Pairwise distribution analysis**				
*R*_g_ (Å)	35.2	36.3	32.8	33.3
*I*(0) (cm^−1^)	0.03	0.01	0.01	0.01
*d*_max_ (Å)	116.5	114.3	99.0	100.2
*V*_p_ (Å^3^)	139000	153000	134000	170000
*q* range (Å^−1^)	0.012–0.20	0.013–0.20	0.013–0.20	0.012–0.020
**SAXS MoW estimate**				
*M_W_* (Da)	Not determined	124000	85000	95000

For the SAXS data collected using an injection concentration of 8.0 mg.ml^−1^ (180 μM), *Pae*DAH7PS^PA1901^ eluted as a single peak with a trailing back edge, indicating polydispersity in the sample. The scattering data were deconvoluted using the HPLC module of the *SOMO* package through the fitting of Gaussian functions to the SEC-SAXS data [52,55,57]. The analysis indicated that there were at least two protein populations contributing to the single elution peak of the SEC-SAXS data. Two pure Gaussian functions were applied to the data, resulting in two distinct scattering profiles; peak A and peak B. Peak A represents the front edge of the elution peak (*R_g_* = 36.0 ± 1.2 Å, *d*_max_ = 114 Å) while peak B was found to spread across the entire elution peak (*R_g_* = 33.0 ± 1.4 Å, *d*_max_ = 99 Å). The calculated *d*_max_ values from the crystal structure of *Pae*DAH7PS^PA1901^ (PDB: 6BMC) for the tetramer, dimer, or monomer are 115.5, 93.3, or 62 Å respectively, with the calculated *d*_max_ values for peaks A and B more closely resembling that determined from the tetrameric or dimeric crystal structures of *Pae*DAH7PS^PA1901^ respectively. In addition, the calculated *R*_g_ values from the crystal structure of *Pae*DAH7PS^PA1901^ for the tetrameric, dimeric, or monomeric species are 39.2, 29.2, and 20.9 Å respectively, with the calculated *R*_g_ values for peaks A and B more closely resembling those determined from the tetrameric or dimeric crystal structures of *Pae*DAH7PS^PA1901^ respectively. Estimated molecular weights for peaks A and B were calculated using SAXS MoW, which is typically accurate within ±10% [72]. The estimated molecular weights for peaks A and B were 124.5 and 84.6 kDa respectively and are comparable, albeit slightly smaller, with the expected molecular weights for the tetrameric or dimeric *Pae*DAH7PS^PA1901^ of 177.88 and 88.94 kDa respectively.

**Figure 9:**
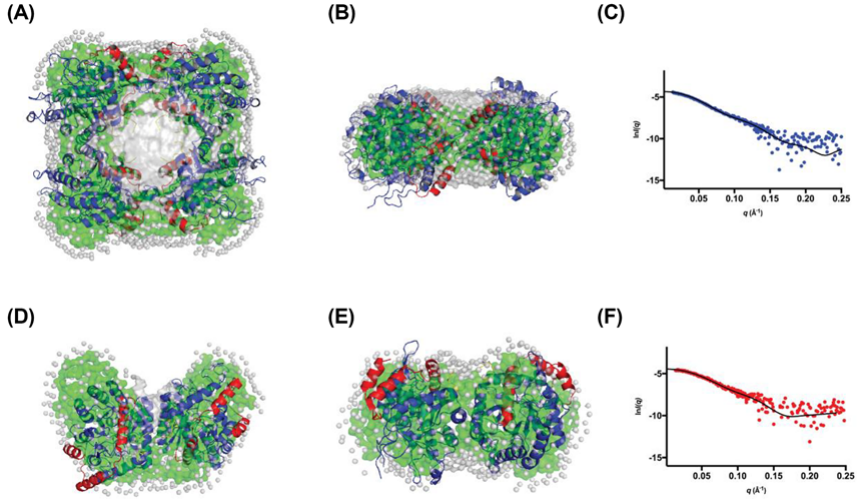
*GASBOR* modelling results for *Pae*DAH7PS^PA1901^ (**A**) *GASBOR* bead model, generated using the *P*(*r*) from peak A (8.0 mg.ml^−1^), with the tetrameric crystal structure of *Pae*DAH7PS^PA1901^ overlaid. (**B**) Side view of the model in (A). (**C**) The fit of the *ab initio* bead model (black line) in (A,B) to the experimental SAXS data (blue circles) from peak A. (**D**) *GASBOR* bead model, generated using the *P*(*r*) from peak B, with the dimeric crystal structure of *Pae*DAH7PS^PA1901^ overlaid. (**E**) Side view of the model in (D). For all frames, the core (βα)_8_ catalytic barrel is shown in blue, the N-terminal extension (residues 1–59) is shown in red, the loop α_2_β_3_ is shown in yellow. The *GASBOR* model is represented by the green surface and modelled solvent molecules are represented in grey. (**F**) The fit of the model (black line) in (D,E) to the experimental SAXS data (red circles) generated from peak B (8.0 mg.ml^−1^).

*Ab initio* bead models (*GASBOR*) were generated from the deconvoluted scattering profiles obtained for both peaks A and B to reconstruct the solution-state tetrameric and dimeric species of *Pae*DAH7PS^PA1901^ and to compare the resultant bead models with the oligomeric assemblies observed in the crystal structure (PDB: 6BMC) (Figure 9). Comparison between the theoretical scattering profiles calculated from the *ab initio* models and the deconvoluted experimental data (Figure 9C,F) suggests that the *ab initio* models are representative of the solution-state tetrameric and dimeric species of *Pae*DAH7PS^PA1901^, which are remarkably similar to those observed in the crystal structure.

**Figure 10:**
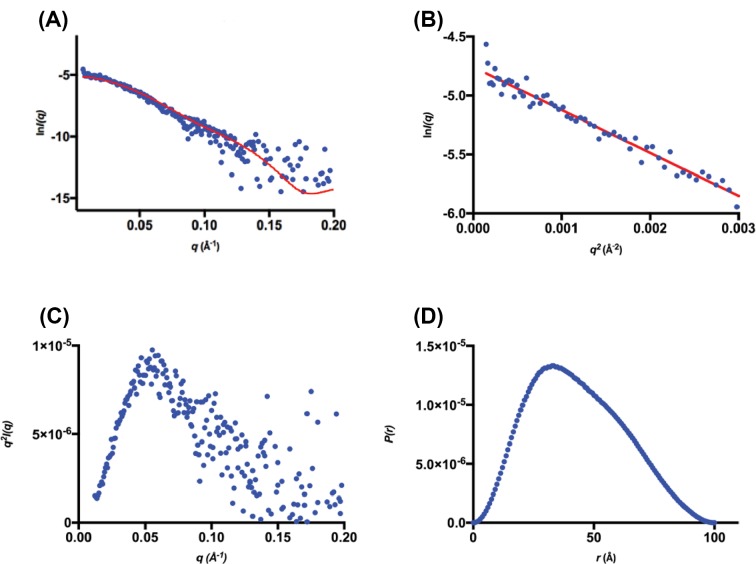
Analysis of SEC-SAXS results obtained for *Pae*DAH7PS^PA1901^ Using a 1.0 mg.ml^−1^ injection concentration. (**A**) log *I(q)* compared with *q*, error bars are indicated in grey, with the theoretical scattering profile calculated from the crystallographic dimer (PDB: 6BMC) overlaid (red line). (**B**) Guinier plot (*ln(I)* compared with *q^2^*). (**C**) Kratky plot (*q^2^⋅I(q)* compared with *q*) for the data in (A). (**D**) *P(r)* compared with *r* profiles for the data in (A).

Due to the decreased signal-to-noise ratio for the SEC-SAXS data collected using an injection concentration of 1.0 mg.ml^−1^ (22 μM), deconvolution of this dataset was not attempted. CRYSOL analysis of the SEC-SAXS data, collected using an injection concentration of 1.0 mg.ml^−1^, indicates that the enzyme exists primarily in the dimeric form (χ^2^ = 0.31 for the fit of the dimeric crystal structure PDB: 6BMC to the experimental data, Figure 10). The *d*_max_ value determined from the 1.0 mg.ml^−1^ SEC-SAXS data of 100.2 Å is consistent with the *d*_max_ value determined either from the dimeric crystal structure of *Pae*DAH7PS^PA1901^ (93.3 Å) or for the deconvoluted peak B (99.0 Å). In addition, the SAXS MoW estimated molecular weight of 95.0 kDa from this low concentration SEC-SAXS data is in close agreement, albeit slightly larger, with the value estimated from the deconvoluted peak B (84.6 kDa) and the expected molecular weight for dimeric *Pae*DAH7PS^PA1901^ (88.94 kDa). The SEC-SAXS parameters determined for the data collected using an injection concentration of 1.0 mg.ml^−1^, in combination with those determined for the deconvoluted 8.0 mg.ml^−1^ data, show that *Pae*DAH7PS^PA1901^ exists in a concentration-dependent equilibrium that favours the dimeric form on decreasing enzyme concentration.

**Figure 11:**
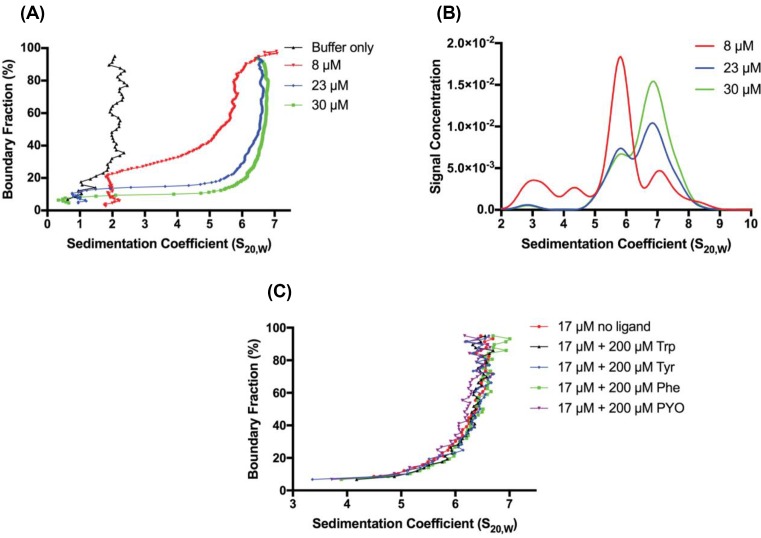
Sedimentation velocity data obtained for *Pae*DAH7PS^PA1901^ (**A**) van Holde–Weischet distributions for *Pae*DAH7PS^PA1901^ at three concentrations (8, 23 and 30 μM) show a shift of the distributions to the right with increasing concentration. (**B**) Combined *S*_20,w_ distribution plots from 2DSA-Monte Carlo analysis reveal major species between 5.8 and 6.8 S. (**C**) van Holde–Weischet analysis of *Pae*DAH7PS^PA1901^ (17 μM) indicates no significant change in the oligomeric state of the protein the presence of either 200 μM of PYO, Phe, Tyr or Trp.

Analytical ultracentrifugation (AUC) experiments carried out at enzyme concentrations ranging from 0.34 to 1.35 mg.ml^−1^ (8–30 μM) were used to confirm the oligomeric state of *Pae*DAH7PS^PA1901^ in solution. Analyses of the absorbance data, collected in intensity mode, by van Holde–Weischet analysis reveal half-parabola shaped s-distributions, which shift to the right (Figure 11A) upon increasing protein concentration, suggesting an interacting, reversible system [50]. Non-interacting species between 1–2 S are likely sedimenting buffer components, as illustrated by analysis of buffer without protein present (Figure 11A). 2DSA-Monte Carlo sedimentation coefficient distributions reveal species with sedimentation coefficients between 5.8 and 6.8 S (Figure 11B), consistent with a molecular weight in the range of 70–96 kDa (Supplementary Figure S6), suggesting that at these concentrations, *Pae*DAH7PS^PA1901^ exists predominantly as a homodimer. Species at ∼3 S, present in the 8 μM distribution (collected at 240 nm), are likely buffer components that absorb at wavelengths lower than 280 nm, as these species are also present in distributions (also collected at 240 nm) of buffer without protein (data not shown), and to a lesser extent in the 11, 23, and 30 μM samples (Figure 11B). A bead model based on the dimeric crystal structure of *Pae*DAH7PS^PA1901^ (PDB: 6BMC) was created with US-SOMO and used to calculate a theoretical sedimentation coefficient of 5.5 S, further suggesting that the species observed for *Pae*DAH7PS^PA1901^ is primarily dimeric. Additional sedimentation velocity experiments, carried out in absorbance mode in the presence of 200 μM of either PYO, Phe, Tyr or Trp, and analysed by van Holde–Weischet analysis, indicate that the presence of either PYO or aromatic amino acids does not influence the oligomeric state of the protein (Figure 11C).

While the formation of a tetrameric species for *Pae*DAH7PS^PA1901^ is observable both in the crystal structure and in solution by SAXS at high injection concentrations (112–180 μM), the nature of the alternative minor interface (and lack of hydrophobic interactions), in combination with the observation of a primarily dimeric species by AUC at protein concentrations less than 30 μM, suggests that at physiological concentrations *Pae*DAH7PS^PA1901^ predominantly persists in the dimeric form. The observation of higher-order solution-state species by SEC-SAXS appears to be the consequence of high enzyme concentration.

### Evolutionary implications

The structural similarities between the N-terminal extensions (helices α_0a_, α_0b_ and α_0c_) found in *Pae*DAH7PS^PA1901^, *Pae*DAH7PS^PA2843^ or *Mtu*DAH7PS, suggest a common origin for this structural element in the type II DAH7PSs. The distinct functionalities of the N-terminal extension within these three enzymes (burying a hydrophobic surface or interface formation for the delivery of allosteric binding sites or combinations thereof), coupled with the physiological roles of these enzymes within primary or secondary metabolism, indicate an evolutionary divergence. The evolutionary trajectory for the type II DAH7PSs appears to have diverged to deliver both an unregulated dimeric group of type II DAH7PSs, suitable for a role within secondary metabolism, and a regulated tetrameric group of type II DAH7PSs that functions within primary metabolism.

For the type II DAH7PSs from *P. aeruginosa*, direct control of enzymatic activity by pathway end products appears largely superfluous as genetic level regulation may be better suited to differentially regulate the expression of multiple DAH7PSs, that function within primary or secondary metabolism, where the presence of aromatic amino acids acts to divert metabolic flux away from primary metabolism and towards the biosynthesis of PCA and its derivatives. Under these conditions, the DAH7PSs that are involved directly within primary metabolism would likely be allosterically inhibited by Trp, Tyr or Phe and thus unavailable to provide chorismate to support the biosynthesis of secondary aromatic metabolites. The presence of *Pae*DAH7PS^PA1901^ within the *phzA–G* biosynthetic cluster allows for the synchronised expression of these proteins required for PCA production. The omission of the α_2a_ and α_2b_ helices in *Pae*DAH7PS^PA1901^, and subsequent insensitivity to allosteric inhibition by Trp, Tyr or Phe, allows for the continued production of chorismate under conditions of high aromatic amino acids, consistent with the alternative, dimeric solution-state structure observed for *Pae*DAH7PS^PA1901^.

## Conclusion

The structure of *Pae*DAH7PS^PA1901^ further highlights the complex evolutionary trajectory for the type II DAH7PSs that has delivered type II enzymes which exhibit a diverse range of quaternary assemblies, and associated allosteric functionalities, required to support the efficient production of chorismate within either primary or secondary metabolism. *Pae*DAH7PS^PA1901^ adopts a dimeric solution-state structure, unlike any other quaternary association observed for the DAH7PSs characterised to date. Surprisingly, *Pae*DAHPS^PA1901^ contains a novel major interface that has not previously been characterised in any DAH7PS. The formation of this alternative major interface in *Pae*DAH7PS^PA1901^, relative to either of the oligomeric interfaces observed in *Pae*DAH7PS^PA2843^ or *Mtu*DAH7PS, disrupts completely the formation of any aromatic amino acid allosteric binding sites that are comparable with those observed in *Pae*DAH7PS^PA2843^ or *Mtu*DAH7PS. The subsequent insensitivity of *Pae*DAH7PS^PA1901^ to allosteric inhibition by aromatic amino acids is compatible with delivering chorismate to support secondary metabolism, in contrast with *Pae*DAH7PS^PA2843^ or *Mtu*DAH7PS, which are sensitive to either Trp or combinations of aromatic amino acids that include Trp, and function primarily within primary metabolism.

Clear sequence diversity exists between the two type II DAH7PS groups identified by sequence clustering analysis. These different sequence characteristics translate directly into two groups of type II DAH7PSs that form significantly different oligomeric interfaces and quaternary assemblies with associated distinct allosteric functionalities. In addition, these differences in quaternary assembly and allosteric behaviour between the two type II DAH7PS groups relate to their defined physiological roles within either primary or secondary metabolism. On this basis, we propose that there is sufficient diversity between these two groups of type II DAH7PSs, both in terms of primary structure and functionality of the resultant enzymes, that the type II DAH7PSs be further categorised as type II_A_ and type II_B_. The type II_A_ DAH7PSs comprise full-length enzymes containing both an N-terminal extension and the α_2a_ and α_2b_ helices (for example *Pae*DAH7PS^PA2843^, *Mtu*DAH7PS or *Cgl*DAH7PS). Type II_A_DAH7PS function primarily within primary metabolism, whereas the type II_B_ DAH7PSs comprise short-form enzymes that contain the N-terminal extension but omit the α_2a_ and α_2b_ helices and these function primarily within secondary metabolism (for example *Pae*DAH7PS^PA1901^).

## Supporting information

**Figure S1:** **Sequence Alignment among the type II DAH7PSs**, indicating the relative positions of β strands and α helices based on *Mtu*DAH7PS (PDB 2B7O). *Mtu, Mycobacterium tuberculosis; Pae*_PA2843, *Pseudomonas aeruginosa; Pae*_PA1901, *Pseudomonas aeruginosa; Sac, Streptomyces achromogens; Pch, Pseudomonas chlororaphis; Sfu*_SFUL3326, *Streptomyces fulvissimus; Sfu*_SFUL5264, *Streptomyces fulvissimus*.

**Figure S2:** **Activity of *Pae*DAH7PS^PA1901^ measured at a range of pH, relative to the activity observed at pH 7.5**, indicates that this enzyme is active over a range of pH between pH 6.5 and 7.5. Substrate concentrations were held at 150 μM each for PEP and E4P.

**Figure S3:** **The melting temperature of *Pae*DAH7PS^PA1901^ and comparison to that previously reported for *Pae*DAH7PS^PA2843^** [1] in the presence of various combinations of aromatic amino acids. Each single letter code corresponds to 200 μM of the appropriate amino acid.

**Figure S4:** **Electron density omit maps for PEP at the active site of *Pae*DAH7PS^PA1901^ (PDB 6BMC).** 2|Fo|-|Fc| is represented by the blue mesh contoured at 1.5 σ and |Fo|-|Fc| is represented by the green mesh contoured at 3 σ.

**Figure S5:** **SEC-SAXS analysis for *Pae*DAH7PS^PA1901^ from the 5.0 mg/mL SEC-input concentration.** (A) Deconvolution of the SEC-SAXS data indicates two Gaussian components (peak A, blue line and peak B, green line. Sum, red line). The *R*_g_ values across each peak are indicated as magenta or cyan squares respectively. (B) - (C) the SAXS profiles for the deconvoluted data peak A and peak B respectively. Guinier plots are inset. (D) Kratky plots of the deconvoluted data in (B) and (C) (peak A, blue circles and peak B, red squares). (E) P(*r*) plots for the deconvoluted data in (B) and (C) (peak A, blue circles and peak B, red squares).

**Figure S6:** **Pseudo-3D plots for distributions of the 2DSA Monte Carlo results shown in Figure 10** for (A) 8 μM, (B) 23 μM, and (C) 30 μM concentrations of *Pae*DAH7PS^PA1901^.

**Table S1:** SEC-SAXS sample and data collection parameters.

**Table S2:** SEC-SAXS parameters determined for PaeDAH7PS^PA1901^ from the 5.0 mg/mL SEC-input concentration.

**Table S3:** RMSD values obtained for the alignment between the structures of *Pae*DAH7PS^PA1901^ and either *Pae*DAH7PS^PA2843^ or *Mtu*DAH7PS.

## Abbreviations

AUC: analytical ultracentrifugation
*Cgl*: *Corynebacterium glutamicum*
DAH7P: 3-deoxy-d-*arabino*-heptulosonate 7-phosphate
DAH7PS: DAH7P synthase
DMGA: Discrete Model Genetic Algorithm
E4P: erythrose 4-phosphate
*Mtu*: *Mycobacterium tuberculosis*
*Pae*: *Pseudomonas aeruginosa*
PCA: phenazine-1-carboxylic acid
PDB: Protein Data Bank
PEP: phosphoenolpyruvate
Phe: phenylalanine
PYO: pyocyanin
SAXS: small angle X-ray scattering
SEC-SAXS: size-exclusion chromatography coupled SAXS
Tyr: tyrosine
Trp: tryptophan
TEV: tobacco etch virus protease
