# Supplementary material for: Structural and functional characterisation of the entry point to pyocyanin biosynthesis in Pseudomonas aeruginosa defines a new 3-deoxy-d-arabino-heptulosonate 7-phosphate synthase subclass
Source: Biosci Rep. 2018 Oct 15;38(5):BSR20181605. doi: 10.1042/BSR20181605 (PMC6435475; doi:10.1042/BSR20181605)
Supplement: Supplementary file 1 [file bsr20181605_Supp1.pdf]

Structural and Functional Characterisation of the Entry Point to Pyocyanin Biosynthesis in *Pseudomonas aeruginosa* Defines a New 3-Deoxy-D-*arabino*-heptulosonate 7-phosphate Synthase Subclass

Oliver W. Sterritt[1,2], Eric J. M. Lang[1,#], Sarah A. Kessans[1], Timothy M. Ryan[3], Borries Demeler[4], Geoffrey B. Jameson[1,2,5,] and Emily J. Parker[1,2,6,*]*

[1]Biomolecular Interaction Centre and School of Physical and Chemical Sciences, University of Canterbury, Christchurch 8041, New Zealand

[2]Maurice Wilkins Centre for Molecular Biodiscovery, University of Auckland 1142, New Zealand

[3]SAXS/WAXS, Australian Synchrotron/ANSTO, 800 Blackburn Road, Clayton, VIC 3168, Australia

[4]Department of Chemistry and Biochemistry, University of Lethbridge, Alberta T1K 3M4, Canada

[5]Institute of Fundamental Sciences and the Riddet Institute, Massey University, Palmerston North 4442, New Zealand

[6]Ferrier Research Institute, Victoria University of Wellington, Wellington 6012, New Zealand

[#]Present address: School of Chemistry, University of Bristol, Bristol BS8 1TS, United Kingdom

SUPPORTING INFORMATION

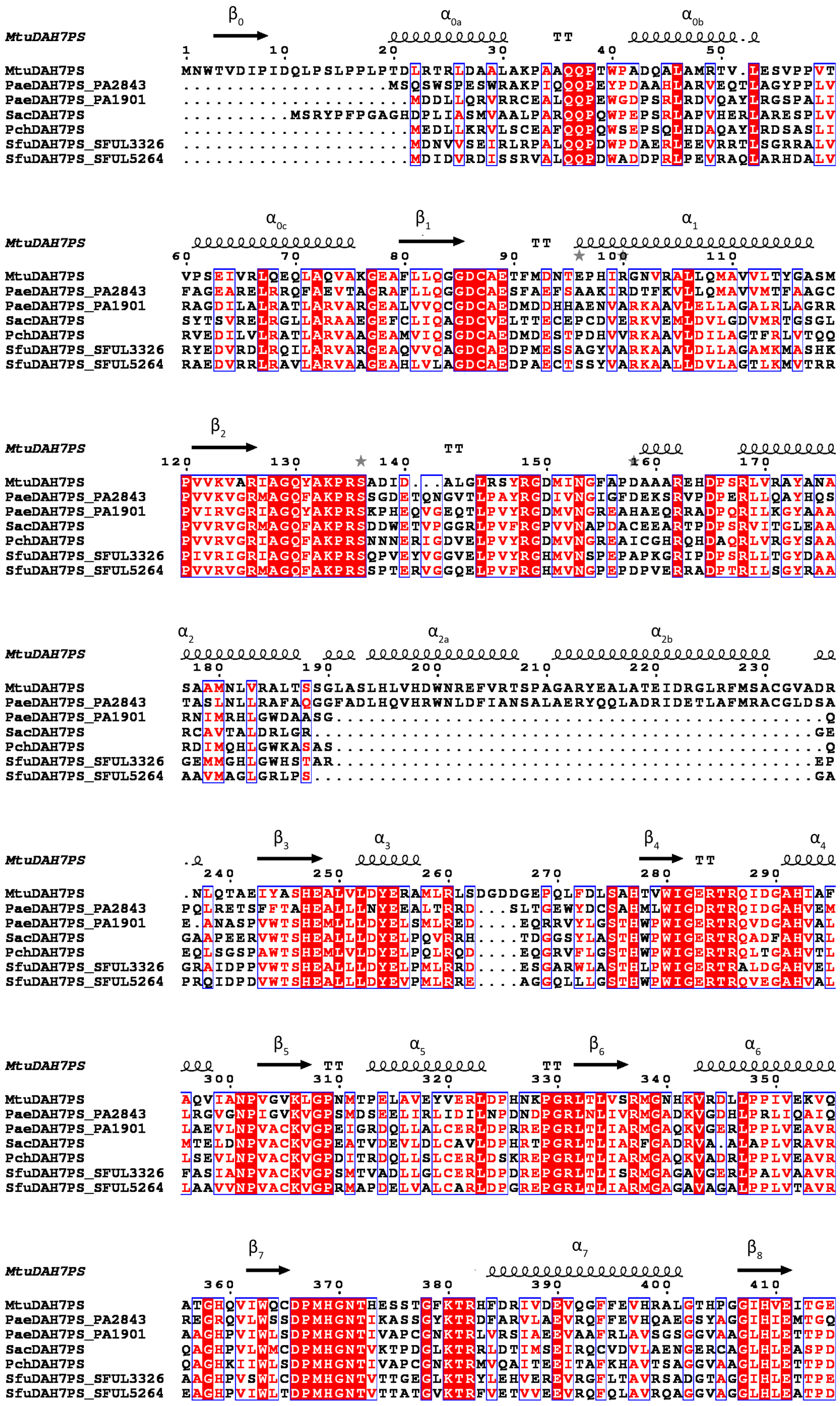
MtuDAH7PS
β0 α0a α0b
1 10 20 30 40 50
MtuDAH7PS MNWTVDIPIDQLPSLPPLPTDLRTRLDAALAKPAAQQPTWPADQALAMRTV.LESVPPVT
PaeDAH7PS_PA2843 ....................MSQSWSPESWRAKPIQQPEYPDAAHLARVEQTLAGYPPLV
PaeDAH7PS_PA1901 ....................MDDLLQRVRRCEALQPEWGDPSRLRDVQAYLRGSPALI
SacDAH7PS ..........MSRYPFPGAGHDPLIASMVAALPARQQPQWPEPSRLAPVHERLARESPLV
PchDAH7PS ....................MEDLLKRVLSCEAFQQPQWSEPSQLHDAQAYLRDSASLI
SfuDAH7PS_SFUL3326 ....................MDNVVSEIRLRPALQQPDWPDAERLEEVRRTLSGRRALV
SfuDAH7PS_SFUL5264 ....................MDIDVRDISSRVALQQPDWADDPRLPEVRAQLARHDALV
MtuDAH7PS
α0c β1 α1
60 70 80 90 100 110
MtuDAH7PS VPSEIVRLQEQLAQVAKGEAFLLQGGDCAETFMDNTEPHIRGNVRALLQMAVVLTYGASM
PaeDAH7PS_PA2843 FAGEARELRRQFAEVTAGRAFLLQGGDCAESFAEFSAAKIRDTFKVLLQMAVVMTFAAGC
PaeDAH7PS_PA1901 RAGDILALRATLARVARGEALVVQCGDCAEDMDDHHAENVARKAAVLELLAGALRLAGRR
SacDAH7PS SYTSVRELRGLLARAAEGEFCLIQAGDCVELTTECEPCDVERKVEMLDVLGDVMRTGSGL
PchDAH7PS RVEDILVLRATLARVAACEAMVIQSGDCAEDMDESTPDHVVRKAAVLDILAGTFRLVTQQ
SfuDAH7PS_SFUL3326 RYEDVRDLRQILARVARGEAQVVQAGDCAEDPMESSAGYVARKAAVLDLLAGAMKMASHK
SfuDAH7PS_SFUL5264 RAEDVRRLRAVLARVAAGEAHLVLAGDCAEDPAECTSSYVARKAALLDVLAGTLKMVTRR
MtuDAH7PS
β2
120 130 140 150 160 170
MtuDAH7PS PVVKVARIAGQYAKPRSADID...ALGLRSYRGDMINGFAPDAAAREHDPSRLVRAYANA
PaeDAH7PS_PA2843 PVVKVGRMAGQFAKPRSSGDETQNGVTLPAYRGDIVNGIGFDEKSRVPDPERLLQAYHQS
PaeDAH7PS_PA1901 PVIRVGRIAGQYAKPRSKPHEQVGEQTLPVYRGDMVNGREAHAEQRRADPQRILKGYAAA
SacDAH7PS PVVRVGRMAGQFAKPRSDDWETVPGGRLPVFRGPVVNAPDACEEARTPDPSRVITGLEAA
PchDAH7PS PVVRVGRIAGQFAKPRSNNNERIGDVELPVYRGDMVNGREAICGHRQHDAQRLVRGYSAA
SfuDAH7PS_SFUL3326 PIVRIGRIAGQFAKPRSQPVEYVGGVELPVYRGHMVNSPEPAPKGRIPDPSRLLTGYDAA
SfuDAH7PS_SFUL5264 PVVRVGRMAGQFAKPRSSPTERVGGQELPVFRGHMVNGPEPDPVERRADPTRILSGYRAA
MtuDAH7PS
α2 α2a α2b
180 190 200 210 220 230
MtuDAH7PS SAAMNLVRALTSSGLASLHLVHDWNREFVRTSPAGARYEALATEIDRGLRFMSACGVADR
PaeDAH7PS_PA2843 TASLNLLRAFAQGGFADLHQVHRWNLDFIANSALAERYQQLADRIDETLAFMRACGLDSA
PaeDAH7PS_PA1901 RNIMRHLGWDAASG...Q
SacDAH7PS RCAVTALDRLGR..GE
PchDAH7PS RDIMQHLGWKASAS...Q
SfuDAH7PS_SFUL3326 GEMMGHLGWHSTAR..EP
SfuDAH7PS_SFUL5264 AAVMAGLGRLPS..GA
MtuDAH7PS
β3 α3 β4 α4
240 250 260 270 280 290
MtuDAH7PS .NLQTAEIYASHEALVLDYERAMLRLSDGDDGEPQLFDLSAHTVWIGERTRQIDGAHIAF
PaeDAH7PS_PA2843 PQLRETSFFTAHEALLLNYEEALTRRD...SLTGEWYDCSAHMLWIGDRTRQIDGAHVEM
PaeDAH7PS_PA1901 E.ANASPVWTSHEMLLLDYELSMLRED....EQRRVYLGSTHWPWIGERTRQVDGAHVAL
SacDAH7PS GAAPEERVWTSHEALLLDYELPQVRRH....TDGGSYLASTHWPWIGERTRQADFAHVRL
PchDAH7PS EQLSGSPAWTSHEMLVLDYELPQLRQD....EQGRVFLGSTHWPWIGERTRQLTGAHVTL
SfuDAH7PS_SFUL3326 GRAIDPPVWTSHEALLLDYELPMLRRD....ESGARWLASTHLPWIGERTRALDGAHVEL
SfuDAH7PS_SFUL5264 PRQIDPDVWTSHEALLLDYEVPMLRRE....AGGQLLLGSTHWPWIGERTRQVEGAHVAL
MtuDAH7PS
β5 α5 β6 α6
300 310 320 330 340 350
MtuDAH7PS AQVIANPVGVKLGPNMTPELAVEYVERLDPHNKPGRLTLVSRMGNHKVRDLLPPIVEKVQ
PaeDAH7PS_PA2843 LRGVGNPIGVKVGPSMDSEELIRLIDILNPDNDPGRLNLIVRMGADKVGDHLPRLIQAIQ
PaeDAH7PS_PA1901 LAEVLNPVACKVGPEIGRDQLLALCERLDPRREPGRLTLIARMGAQKVGERLPPLVEAVR
SacDAH7PS MTELDNPVACKVGPEATVDEVLDLCAVLDPHRTPGRLTLIARFGADRVA.ALAPLVRAVR
PchDAH7PS LSEVLNPVACKVGPDITRDQLLSLCERLDSKREPGRLTLIARMGAQKVADRLPPLVEAVR
SfuDAH7PS_SFUL3326 FASIANPVACKVGPSMTVADLLGLCERLDPDREPGRLTLISRMGAGAVGERLPALVAAVR
SfuDAH7PS_SFUL5264 LAAVVNPVACKVGPRMAPDELVALCARLDPGREPGRLTLIARMGAGAVAGALPPLVTAVR
MtuDAH7PS
β7 α7 β8
360 370 380 390 400 410
MtuDAH7PS ATGHQVIWQCDPMHGNTHESSTGFKTRHFDRIVDEVQGFFEVHRALGTHPGGIHVEITGE
PaeDAH7PS_PA2843 REGRQVLWSSDPMHGNTIKASSGYKTRDFARVLAEVRQFFEVHQAEGSYAGGIHIEMTGQ
PaeDAH7PS_PA1901 AAGHPVIWLSDPMHGNTIVAPCGNKTRLVRSIAEEVAAFRLAVSGSGGVAAGLHLETTPD
SacDAH7PS QAGHPVLWMCDPMHGNTVKTPDGLKTRRLDTIMSEIRQCVDVLAENGERCAGLHLEASPD
PchDAH7PS QAGHKIIWLSDPMHGNTIVAPCGNKTRMVQAITEEITAFKHAVTSAGGVAAGLHLETTPD
SfuDAH7PS_SFUL3326 AAGHPVSWLCDPMHGNTVTTGEGLKTRYLEHVEREVRGFLTAVRSADGTAGGIHLETTPE
SfuDAH7PS_SFUL5264 EAGHPVIWLTDPMHGNTVTTATGVKTRFVETVVEEVRQFQLAVRQAGGVAAGLHLEATPD

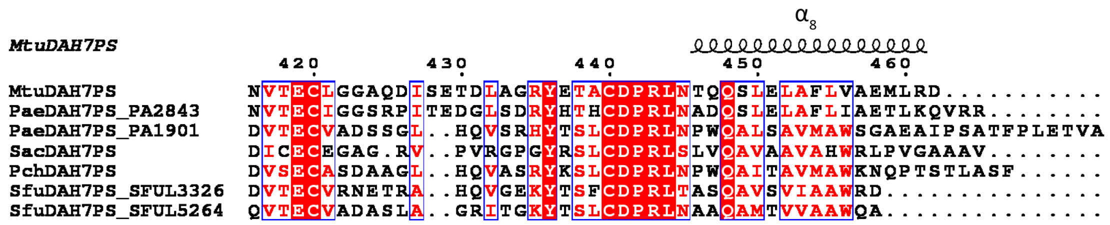

Figure S1: Sequence Alignment among the type II DAH7PSs, indicating the relative positions of β strands and α helices based on *Mtu*DAH7PS (PDB 2B7O). *Mtu*, *Mycobacterium tuberculosis*; *Pae*_PA2843, *Pseudomonas aeruginosa*; *Pae*_PA1901, *Pseudomonas aeruginosa*; *Sac*, *Streptomyces achromogens*; *Pch*, *Pseudomonas chlororaphis*; *Sfu*_SFUL3326, *Streptomyces fulvissimus*; *Sfu*_SFUL5264, *Streptomyces fulvissimus*.

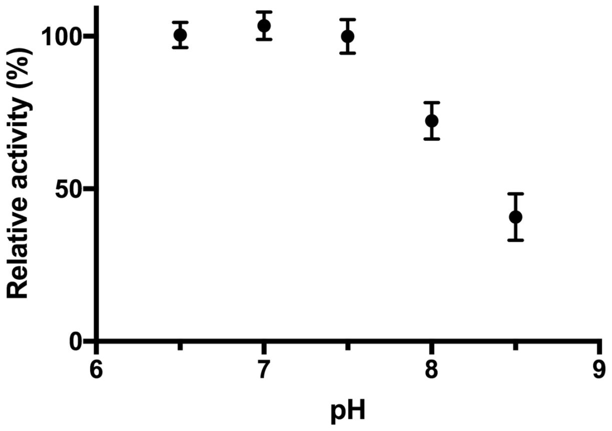

Figure S2: Activity of *Pae*DAH7PS^{PA1901} measured at a range of pH, relative to the activity observed at pH 7.5, indicates that this enzyme is active over a range of pH between pH 6.5 and 7.5. Substrate concentrations were held at 150 μM each for PEP and E4P.

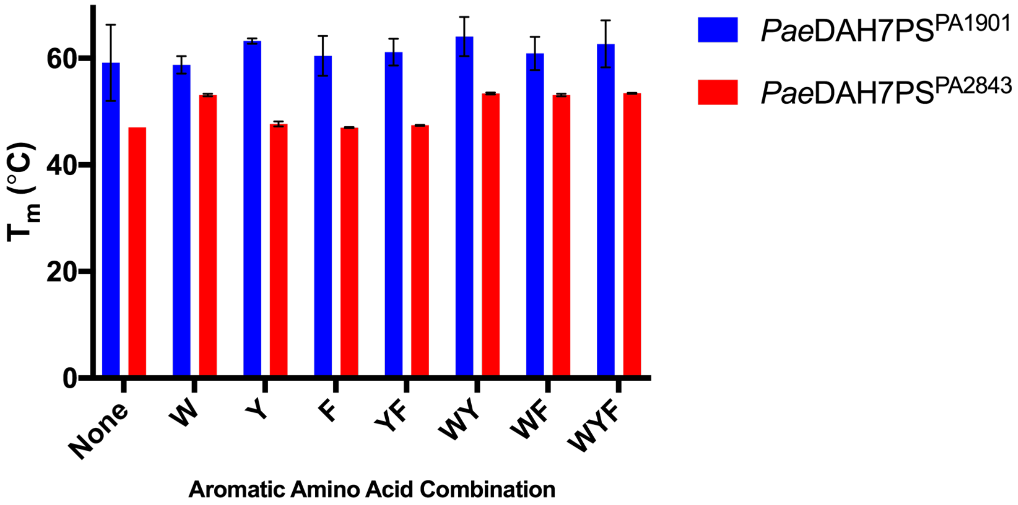

Figure S3: The melting temperature of *Pae*DAH7PS^{PA1901} and comparison to that previously reported for *Pae*DAH7PS^{PA2843} [1] in the presence of various combinations of aromatic amino acids. Each single letter code corresponds to 200 µM of the appropriate amino acid.

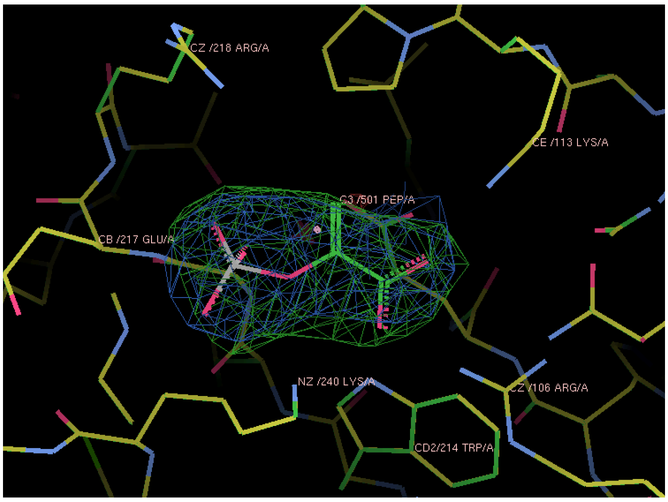

Figure S4: Electron density omit maps for PEP at the active site of *Pae*DAH7PS[PA1901] (PDB 6BMC). 2|Fo|-|Fc| is represented by the blue mesh contoured at 1.5 σ and |Fo|-|Fc| is represented by the green mesh contoured at 3 σ.

Table S1: **SEC-SAXS sample and data collection parameters**.

**Sample Details**	***Pae*DAH7PS^{PA1901}**
Organism	*Pseudomonas aeruginosa*
UniProt sequence ID	G3XCJ9
Extinction coefficient [A_{280}, 0.1 %]	1.22
M from chemical composition (Da)	44, 468
SEC-SAXS column	5 x 150 mm, Superdex S200
Loading concentration (mg mL^{-1})	1.0, 5.0 or 8.0
Injection volume (µL)	20
Flow rate (mL min^{-1})	0.2
Solvent	50 mM bis-Tris propane pH 7.5, 100 µM cobalt chloride, 200 µM PEP, 5 % glycerol
**SAXS data collection parameters**	
Instrument	Australian synchrotron SAXS/WAXS beamline
Detector	Dectris PILATUS 1M
Sample environment	SEC-SAXS with sheath-flow cell
Wavelength (Å)	1.0332
Camera length (mm)	1600
Set temperature (°C)	20

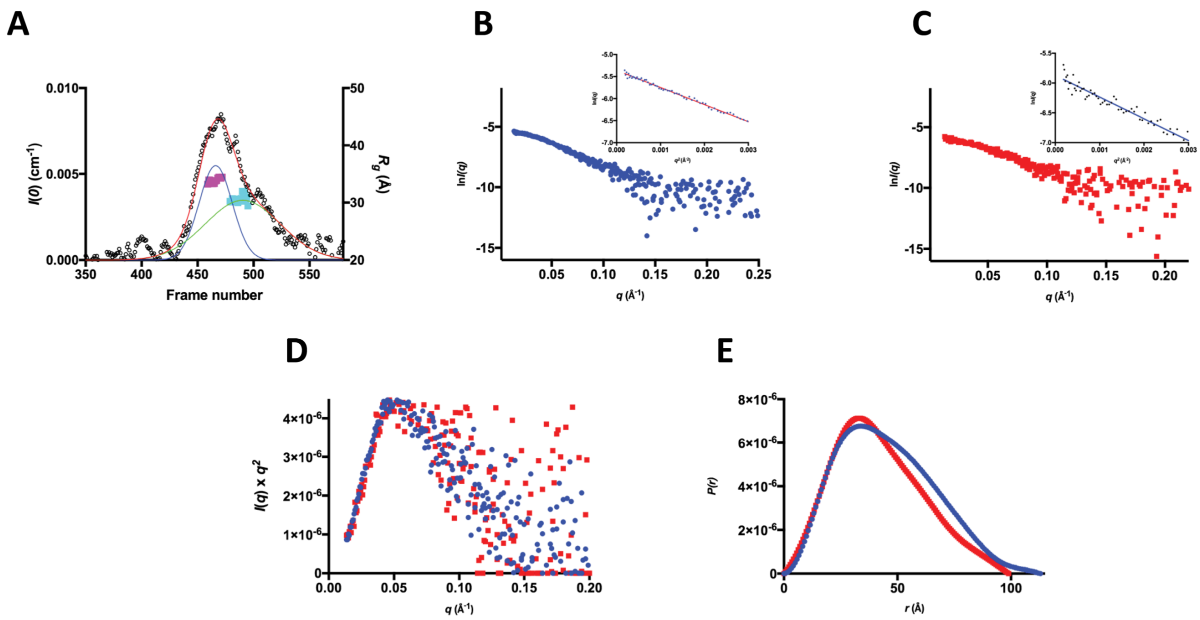

Figure S5: SEC-SAXS analysis for *Pae*DAH7PS^{PA1901} from the 5.0 mg/mL SEC-input concentration. (A) Deconvolution of the SEC-SAXS data indicates two Gaussian components (peak A, blue line and peak B, green line. Sum, red line). The R_g values across each peak are indicated as magenta or cyan squares respectively. (B) – (C) the SAXS profiles for the deconvoluted data peak A and peak B respectively. Guinier plots are inset. (D) Kratky plots of the deconvoluted data in (B) and (C) (peak A, blue circles and peak B, red squares). (E) *P*(*r*) plots for the deconvoluted data in (B) and (C) (peak A, blue circles and peak B, red squares).

Table S2: SEC-SAXS parameters determined for *Pae*DAH7PS^{PA1901} from the 5.0 mg/mL SEC-input concentration.

	5.0 mg/mL Peak A	5.0 mg/mL Peak B
Guinier Analysis		
R_g (Å)	34.0 ± 1.6	32.0 ± 3.8
$I(0)$ (cm^{-1})	0.004 ± $5e^{-5}$	0.004 ± $1e^{-4}$
q_{min}	0.013	0.013
Correlation coefficient, r^2	0.992	0.928
Pair Wise Distribution Analysis		
R_g (Å)	35.25	32.5
$I(0)$ (cm^{-1})	0.00	0.00
d_{max} (Å)	113.1	99.0
V_p ($Å^3$)	167,000	126,000
q range ($Å^{-1}$)	0.013 – 0.25	0.013 – 0.25

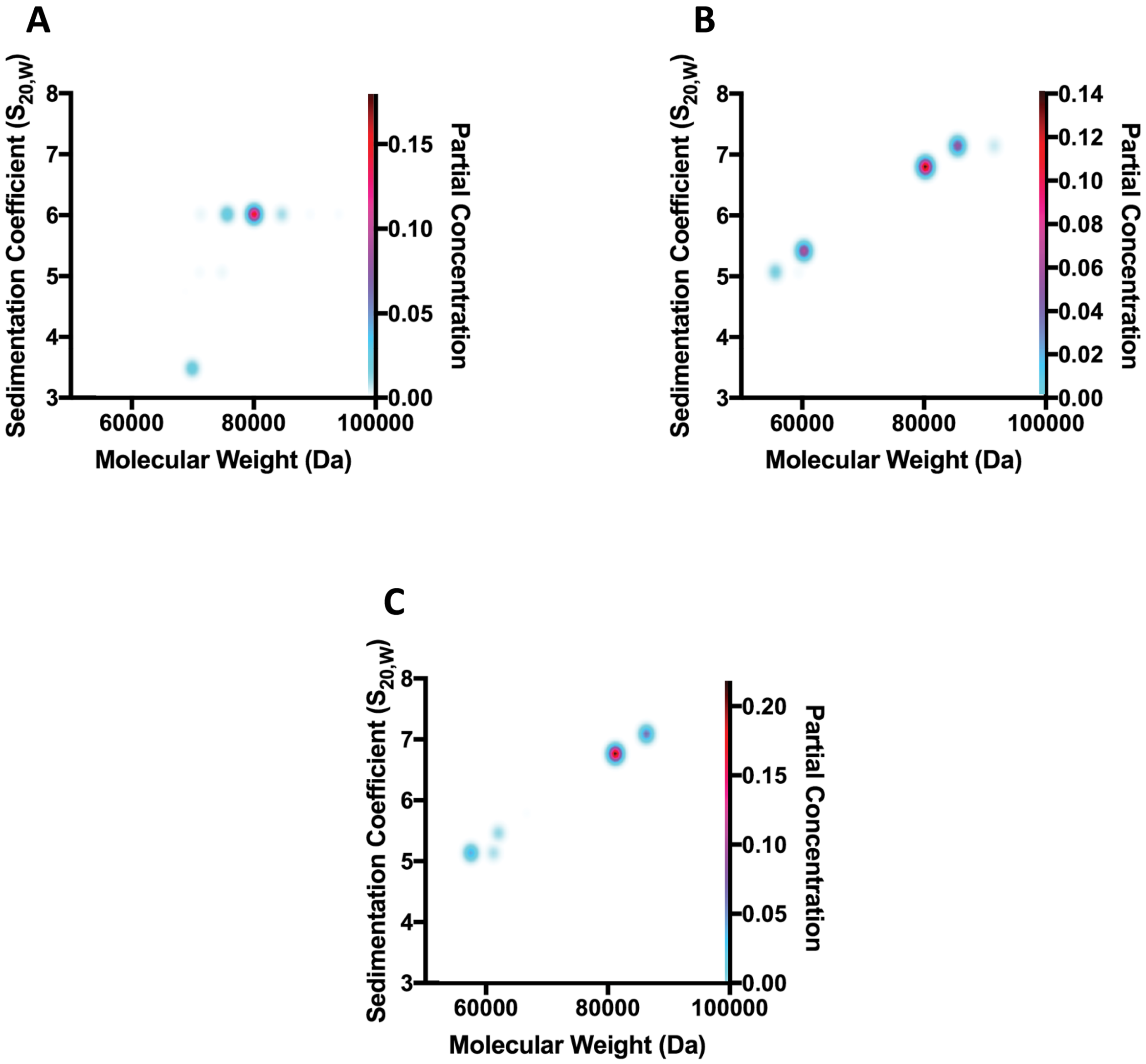

Figure S6: **Pseudo-3D plots for distributions of the 2DSA Monte Carlo results shown in Figure 10** for (A) 8 µM, (B) 23 µM, and (C) 30 µM concentrations of *Pae*DAH7PS^{PA1901}.

Table S3: RMSD values obtained for the alignment between the structures of *Pae*DAH7PS[PA1901] and either *Pae*DAH7PS[PA2843] or *Mtu*DAH7PS.

Alignment between		RMSD (Å)
Single chain		
6BMC_chain A	5UXO	0.816
6BMC_chain A	5UXN	0.830
6BMC_chain A	5UXM	0.822,
6BMC_chain A	2B7O_Chain A	0.775
6BMC_chain A	2B7O_Chain B	0.821
6BMC_chain B	5UXO	0.816
6BMC_Chain B	5UXN	0.817
6BMC_chain B	5UXM	0.840
6BMC_chain B	2B7O_Chain A	0.754
6BMC_chain B	2B7O_Chain B	0.776
Crystallographic Dimer		
6BMC	5UXO	25.740
6BMC	5UXN	26.020
6BMC	5UXM	26.100
6BMC	2B7O	25.100
Crystallographic Tetramer		
6BMC	5UXO	27.850
6BMC	5UXN	28.610
6BMC	5UXM	28.800
6BMC	2B7O	34.460

References

1. Sterritt OW, Kessans, S. A., Jameson, G. B., Parker, E. J. A Pseudoisostructural Type II DAH7PS Enzymes from *Pseudomonas aeruginosa*: alternative evolutionary strategies to control shikimate pathway flux. Biochemistry. 2018;57(18):2667 - 78.
